# Genomic insights into five selected multidrug-resistant *Pseudomonas aeruginosa* isolated from Sodwana Bay, South Africa

**DOI:** 10.3389/fmicb.2025.1578578

**Published:** 2025-07-02

**Authors:** Mahloro Hope Serepa-Dlamini, Kulsum Kondiah, Pfariso Maumela, Abraham Goodness Ogofure, Ezekiel Green

**Affiliations:** ^1^Department of Biotechnology and Food Technology, University of Johannesburg, Doornfontein Campus, Johannesburg, South Africa; ^2^Bacterial Genomics and Biotechnology (BGB) Research Group, Department of Biotechnology and Food Technology, University of Johannesburg, Doornfontein Campus, Johannesburg, South Africa; ^3^Environmental Biotechnology Research Group, Department of Biotechnology and Food Technology, University of Johannesburg, Doornfontein Campus, Johannesburg, South Africa; ^4^Microbial Pathogenicity and Molecular Epidemiology Research Group (MPMERG), Department of Biotechnology and Food Technology, University of Johannesburg, Doornfontein Campus, Johannesburg, South Africa

**Keywords:** *Pseudomonas aeruginosa*, ST244, ST971, whole gene assembly, WGS – whole-genome sequencing, clinical isolates, environmental isolates

## Abstract

*Pseudomonas aeruginosa* (*P. aeruginosa*) is a common environmental organism and the leading cause of opportunistic human diseases. Its inherent tolerance to pharmaceuticals and disinfectants is fundamental to its pathogenicity. This study investigates the genomic characteristics of five multidrug-resistant *Pseudomonas aeruginosa* isolates from Sodwana Bay, South Africa, highlighting resistance profiles and virulence factors. Using Illumina MiSeq sequencing and functional annotation via Prokka, PATRIC and RAST, the analysis revealed significant resistance mechanisms. The isolates clustered with *P. aeruginosa* DSM 50071. The genome sizes for AF, AF1, BIS, BIS1, and BDPW, ranging from 7.0 to 6.4 Mb, were observed, with G + C contents ranging from 66.1 to 66.48%. A *de novo* multi-drug resistance was observed in all the tested strains, while *β*-lactamase resistance genes *blaPAO*, aminoglycoside phosphorylating enzymes genes *aph*(3′)-*IIb*, and fosfomycin resistance gene (*fosA*), were among the resistance genes found in all samples. The ST analysis revealed the presence of high-risk STs (ST244) in the genomes. The *blaOXA*50 gene linked to high-risk STs, which exhibits increased efficacy against carbapenems, was more common in most genomes. The examination of the virulome revealed that the *exoS* was more commonly found in most genomes, whereas the *exoU* was found in only two isolates. This study presents information concerning the genetic heterogeneity among *P. aeruginosa* strains obtained from various habitats and valuable insights into resistance gene distribution in environmental reservoirs.

## 1 Introduction

*Pseudomonas aeruginosa* is an eminently adaptive Gram-negative bacterium and a recognized opportunistic pathogen associated with hospital-acquired infections (HAI) (Verdial et al., 2023). Due to its intrinsic resistance to multiple antibiotic classes, including *β*-lactams, aminoglycosides, and fluoroquinolones, and their formidable biofilm-forming ability, *P. aeruginosa* infections are notoriously difficult to manage (Lister et al., 2009). As a result, the pathogen has been linked to high morbidity and mortality rates, particularly in immunocompromised individuals and those with cystic fibrosis (Oliver et al., 2015). The increasing emergence of multidrug-resistant (MDR), extensively drug-resistant (XDR), and pan-drug-resistant (PDR) *P. aeruginosa* strains further complicates treatment, posing a serious global health threat with limited therapeutic options (Tacconelli et al., 2018).

In South Africa, for example, the increasing prevalence of multidrug-resistant (MDR) *P. aeruginosa* presents a significant public health challenge, particularly in healthcare settings and among vulnerable patient populations. Recent studies underscore the concern about the infection rate due to MDR *P. aeruginosa* across different provinces, highlighting the urgent need for enhanced surveillance and targeted antimicrobial stewardship interventions. A high burden of MDR *P. aeruginosa* has been reported in rural Eastern Cape Province, with isolates predominantly harboring the *blaTEM* and *blaSHV* resistance genes, which confer *β*-lactamase-mediated resistance to broad-spectrum β-lactam antibiotics (Hosu et al., 2021). Moreover, epidemic MDR *P. aeruginosa* strains have also been identified in public cystic fibrosis (CF) clinics across South Africa. The presence of these highly resistant strains necessitates an urgent reconsideration of infection control strategies, including patient segregation and reinforced antimicrobial stewardship, to mitigate nosocomial transmission and prevent potential outbreaks (Hamiwe et al., 2024). Moreover, Ramsamy et al. (2018) assessed the resistance trends in KwaZulu-Natal and contextualized South Africa’s AMR burden within the global priority pathogen framework. Their findings highlight an increasing incidence of infections caused by *P. aeruginosa,* among other ESKAPE pathogens, reinforcing its status as a critical pathogen requiring immediate intervention. In addition to the increasing burden of MDR *P. aeruginosa* infections, the identification of high-risk sequence types (STs) in South Africa further underscores the pathogen’s epidemiological significance. STs are numerical designations assigned to bacterial isolates based on their Multi-Locus Sequence Typing (MLST) profiles (a molecular typing method that characterizes bacterial strains by analyzing the nucleotide sequences of multiple housekeeping genes). Identifying the ST of an isolate provides valuable information about its potential pathogenicity, likely resistance mechanisms, and relationship to globally circulating strains. Notably, the epidemic *P. aeruginosa* AUST-03 (ST242) strain, first reported in Australia in 2003 (Bradbury et al., 2009; Parkins et al., 2018), was found in South African cystic fibrosis (CF) clinics (Hamiwe et al., 2024), raising concerns about intercontinental transmission and persistence within healthcare settings. Beyond ST242, high-risk sequence types (STs) such as ST244—a globally recognized MDR clone—have been identified in Portugal’s hospital environments and wastewater treatment plants (de Sousa et al., 2025). Maclean et al. (2022) also isolated high-risk ST244 and 971 from SCUBA divers with Swimmer’s Ear (*otitis external*), highlighting the adaptability of *P. aeruginosa* to diverse ecological niches. Given the challenges posed by *P. aeruginosa* in clinical settings, it is vital also to investigate its occurrence in non-clinical environments, where it can persist, evolve, and serve as a reservoir for antimicrobial resistance genes. Having been extensively studied in hospitals, increasing attention is now being directed toward its presence in environmental reservoirs, including aquatic systems, soil, and plant surfaces. Its metabolic adaptability, efflux pump-mediated resistance, and ability to acquire exogenous genetic elements—such as antimicrobial resistance (AMR) genes and virulence factors—allow it to thrive in diverse ecological niches (Moradali et al., 2017). Environmental reservoirs are not merely passive habitats but active facilitators of antibiotic resistance evolution and dissemination (Moradali et al., 2017). Recent research indicates that *P. aeruginosa* strains isolated from seas, rivers, and wastewater treatment facilities often exhibit resistance profiles akin to those of clinical isolates, suggesting a potential link between environmental and nosocomial strains (Khan et al., 2008; Aroca-Molina et al., 2024). This raises serious concerns about horizontal gene transfer (HGT), where resistance genes from environmental strains may be transferred to human pathogens, accelerating the spread of antimicrobial resistance (Pirnay et al., 2009). Among environmental reservoirs, marine ecosystems deserve special attention, as they are constantly exposed to diverse selective pressures that may drive the emergence of highly resistant bacterial populations.

The marine environment represents a dynamic and unique ecological system where bacteria are exposed to selective pressures, including naturally occurring antibiotics, heavy metals, and industrial pollutants, which can contribute to antimicrobial resistance development (Martinez, 2009). Coastal areas such as Sodwana Bay, South Africa, experience significant human activity, including tourism, wastewater discharge, and aquaculture. These activities introduce antibiotic residues and resistant bacteria into the marine ecosystem (Hernandez et al., 2019; Gonzalez-Gaya et al., 2022). Despite the increasing recognition of marine environments as reservoirs of antibiotic-resistant bacteria, research on the genomic diversity and resistance mechanisms of *P. aeruginosa* in these settings remains scarce. Without a clear understanding of how environmental *P. aeruginosa* evolve and acquire resistance, assessing their potential impact on human health and clinical outbreaks remains challenging. To bridge this knowledge gap, advances in genomic technologies have become essential, providing a deeper understanding of how *P. aeruginosa* adapts and spreads in different environments.

Advances in whole-genome sequencing (WGS) have significantly enhanced the characterization of *P. aeruginosa* isolates with regards to tracking high risk clones and antimicrobial resistance. For example, Stribling et al. (2024) opined that over 5,000 *P. aeruginosa* isolates had been sequenced and deposited in public databases like the Multi-Drug Resistant Organism Repository and Surveillance Network (MRSN) collection alone with more than 500 high risk clones. However, environmental isolates, particularly from marine environments, remain underrepresented. Whole-genome sequencing (WGS) has proven indispensable in elucidating the genetic composition, resistance factors, and evolutionary adaptations of *P. aeruginosa* (Wee et al., 2018). While WGS studies on clinical isolates have provided critical insights into molecular resistance mechanisms, environmental isolates remain understudied despite their potential role in resistance gene dissemination (Breidenstein et al., 2011). Recent evidence suggests that environmental *P. aeruginosa* strains may harbour novel resistance genes, mobile genetic elements, and virulence factors that could enhance their survival and contribute to the emergence of high-risk clones in clinical settings (Chen et al., 2014; Aroca-Molina et al., 2024). However, genetic surveillance of environmental *P. aeruginosa* remains particularly limited in marine ecosystems, where the mechanisms driving antibiotic resistance evolution are poorly understood. Given these gaps in knowledge, our study aims to explore the genomic characteristics of marine *P. aeruginosa* isolates, particularly their resistance determinants and phylogenetic links to known clinical strains.

We were basically motivated by cases of otitis externa among scuba divers in Sodwana Bay, South Africa, where infections were frequently unresponsive to conventional antibiotics. To investigate potential aquatic environmental sources of multidrug-resistant *P. aeruginosa*, we conducted genomic analyses on isolates obtained from marine water, swimming pool water, and affected divers. Therefore, our study sought to fill this research gap by employing whole-genome sequencing to analyze five *P. aeruginosa* isolates associated with Sodwana Bay aquatic environments. Our objectives include identifying resistance genes, virulence factors, sequence types (STs), and phylogenetic relationships with known high-risk clones. By comparing these isolates to previously characterized clinical strains, this research provides essential data on the role of aquatic environments in antimicrobial resistance dissemination. Understanding the genetic attributes of *P. aeruginosa* from both marine and human-associated aquatic settings is critical for assessing public health implications and developing strategies to mitigate the spread of multidrug-resistant pathogens.

## 2 Materials and methods

### 2.1 Isolation, identification and selection of bacterial strains

The five strains evaluated in the study were classified based on the environmental sources of isolation. Particularly, the isolates (*Pseudomonas* sp. AF1, BIS, AFW1, BIS1, and BDPW) were environmental isolates from Sodwana Bay, South Africa, with AF1 and AFW1 from ocean sediment samples, BIS from a clinical case of *otitis externa* from an infected scuba diver, BIS1 from seawater, and BDPW from a swimming pool sample as reported in our previous study (Maclean et al., 2022). Standard-based culture techniques were employed for the isolation of *Pseudomonas* sp. For the isolation, samples were inoculated on Nutrient agar and *Pseudomonas* cetrimide agar (Oxoid, United Kingdom), and incubated at 37°C for 24–48 h under aerobic conditions. Colonies exhibiting characteristic fluorescent green pigmentation indicative of *P. aeruginosa* were selected (Bridson, 2006). For molecular identification, universal 16S rRNA primers F: GACGGGTGAGTAATGCCTA and R: CACTGGTGTTCCTTCCTATA were used for genus-level identification (Weisburg et al., 1991), and species-specific primers for *P. aeruginosa* F: GGGGGATCTTCGGACCTCA and R: TCCTTAGAGTGCCCACCCG were adopted as described by Spilker et al. (2004). The strains were selected based on their multi-drug resistance profiles (following antibiotic susceptibility) and the different sample sources from our previous study (Maclean et al., 2022). Due to the data-intensive and resource-heavy nature of whole-genome sequencing (WGS), the study was restricted to five isolates to enable in-depth genomic characterization, providing high-resolution insights into resistance genes, virulence factors, and sequence types, without compromising data quality.

### 2.2 Isolation of DNA, sequencing, pre-processing, genome assembly, and annotation

Prior to genomic DNA extraction, isolates were grown overnight in Luria–Bertani (LB) broth at 37°C with shaking at 180 rpm to obtain sufficient biomass. Genomic DNA was extracted from the isolates using the Nucleospin Microbial DNA Kit (Macherey-Nagel, Germany) according to the manufacturer’s instructions. The concentration and quality of the extracted DNA were assessed using a NanoDrop ND-2000 UV–Vis spectrophotometer (Thermo Fisher Scientific, United States). The OD260/280 ratios of the extracted DNA ranged from 1.82 to 1.89, indicating high purity suitable for downstream sequencing. Sequencing was performed on an Illumina MiSeq platform with 300 bp paired-end reads to ensure comprehensive genome coverage. All pre-annotation analyses were conducted on the Galaxy web platform^1^ (Afgan et al., 2016; Galaxy, 2022). Raw reads were first evaluated using FastQC v0.69 to assess per-base sequence quality, GC content, and overrepresented sequences (Andrews, 2010). Reads with quality scores below Q20 (Q < 20) and those containing adapter sequences were trimmed using Trimmomatic v0.39 (Bolger et al., 2014) prior to *de novo* assembly. On average, 1.2 to 2.5 million paired-end reads per sample were generated by the Illumina MiSeq platform (2 × 300 bp). Using default parameters, the sequence reads were de novo assembled using Unicycler v 0.4.1.1 (Wick et al., 2017) and assessed with Quast v 4.6.3 (Gurevich et al., 2013). The assembled genomes demonstrated high contiguity, with N50 values exceeding 50,000 bp, thereby meeting standard quality thresholds for bacterial genome annotation workflows. The draft genome sequence was submitted to NCBI and annotated using the Prokaryotic Genome Annotation Pipeline (PGAP) (Tatusova et al., 2016) and the Rapid Annotations using Subsystems Technology (RAST) server (Aziz et al., 2008; Overbeek et al., 2014), with accession numbers SAMN17601188, SAMN17601189, SAMN17601190, SAMN17601191, and SAMN17601192.

### 2.3 Multilocus sequence typing

Multilocus sequence typing (MLST) was performed to determine the sequence types (STs) of the identified *P. aeruginosa* isolates. The seven housekeeping genes (*acsA, aroE, guaA, mutL, nuoD, ppsA,* and *trpE*) were amplified using standard primers and PCR conditions following manufacturer’s instructions. Allelic profiles and sequence types (STs) were assigned by querying the *P. aeruginosa* MLST database.^2^

### 2.4 Phylogenetic analysis

Whole-genome phylogenetic analysis was performed to determine the evolutionary relationships among the five *P. aeruginosa* isolates and reference strains. Genome sequences were compared based on orthologous proteins using OrthoFinder (Emms and Kelly, 2019), which identifies conserved orthologous sequences across genomes and provides robust phylogenetic reconstructions. Phylogenetic trees were inferred using the Maximum Likelihood method as pairwise genome comparisons were conducted using FastME 2.1.6.12 (Guindon and Gascuel, 2003; Lefort et al., 2015) based on Genome Blast Distance Phylogeny (GBDP) distances computed from the genome sequences. The branch lengths are proportional to GBDP distances (formula d5). Support values above branches represent GBDP pseudo-bootstrap values (>60%) from 100 replicates, with an average support of 95.3%. Trees were rooted at the midpoint (Lemoine et al., 2018) and visualized using FigTree v1.4.4. The metadata regarding strain origin were incorporated as aforementioned in the earlier methodology section. Public reference strains were selected based on their availability and genomic completeness in databases such as NCBI and to cover a broad diversity of the *Pseudomonas* genus, ensuring inclusion of both closely related and more distant species to accurately resolve phylogenetic relationships. Average Nucleotide Identity (ANI) calculations between the isolates were also evaluated using FastANI or pyANI, indicating high genomic relatedness among the isolates.

### 2.5 Bioinformatics

The TYGS (Type Strain Genome Server) study was conducted using the Orthologous Average Nucleotide Identity Tool (OAT) (Lee et al., 2016) software at https://tygs.dsmz.de/ (Wattam et al., 2017) and OrthoANI (Orthologous Average Nucleotide Identity Tool). Screening for specialty genes (antibiotic resistance genes, virulent factors) was done using the Patric website^3^ (Wattam et al., 2017) of the PGAP annotation file produced from the NCBI revealed the Genomic Island (GI). The CRISPR-Cas Finder Software predicted the regularly interspaced Clustered short palindrome repetitions (CRISPR) (Grissa et al., 2007; Wheatley and MacLean, 2021; Gomez-Martinez et al., 2023; Parra-Sanchez et al., 2023). The RAST server was used to record and categorize predicted genes by function. A comparison analysis of *Pseudomonas* common and unique genes was carried out (Aziz et al., 2008; Overbeek et al., 2014). *Pseudomonas* isolates’ common and distinct genes have been evaluated using EDGAR2.0 (Blom et al., 2016), compared to the *P. aeruginosa* POA1 genome. Resistome analysis was carried out, following the instructions delineated by Afgan et al. (2016).

### 2.6 Statistical analysis and data visualization

Data analysis and visualization were conducted using R software (version 4.3.3), employing a robust combination of the *ComplexHeatmap*, *reshape circlize*, *grid*, and *tidyverse packages* (R-Core Team, 2023). The *ComplexHeatmap* package was pivotal for generating high-resolution heatmaps incorporating hierarchical clustering of isolates and bespoke colour scales. Genes were represented using a binary colour scheme to ensure clear differentiation. Additionally, the *rowAnnotation()* function was utilized to display Shared Type (ST) values alongside the isolate names, with unique colour coding for different ST values. The visualization strategy adopted in this study aligns with recognized methodologies from previous research by Ogofure et al. (2024), Ogofure and Green (2025), and Ogofure et al. (2025), which demonstrated the effectiveness of bespoke heatmaps in conveying complex microbiological datasets. Special attention was given to the placement of isolate names, dendrogram arrangement, annotation layout, and label scaling to ensure both interpretability and aesthetic clarity. The comprehensive investigation of genomic diversity across eight *P. aeruginosa* isolates (our five strains (AF1, AFW1, BIS, BIS1, and BDPW) and three control strains (Clinical strain PAO1, reference pathogenic strain ATCC700888 and N002 strain from soil environment)) were visualized through a multi-panel heatmap. The analysis focused on four key genetic elements: resistome, prophage content, CRISPR-Cas systems, and virulence factors. For statistical assessment, we employed a descriptive comparative analysis rather than hypothesis testing by calculating Jaccard similarity indices between isolate pairs for each genetic category to quantify genomic relatedness. Hierarchical clustering analysis using binary distance and average linkage was performed to identify isolate groupings based on their combined genetic profiles. Additionally, we assessed correlations between different genetic element categories using Pearson correlation and calculated feature richness statistics to determine the distribution patterns of genetic elements across isolates.

## 3 Results

### 3.1 Molecular identification, sequencing and phylogenetic relatedness of *Pseudomonas aeruginosa* isolates

**Figure 1:**
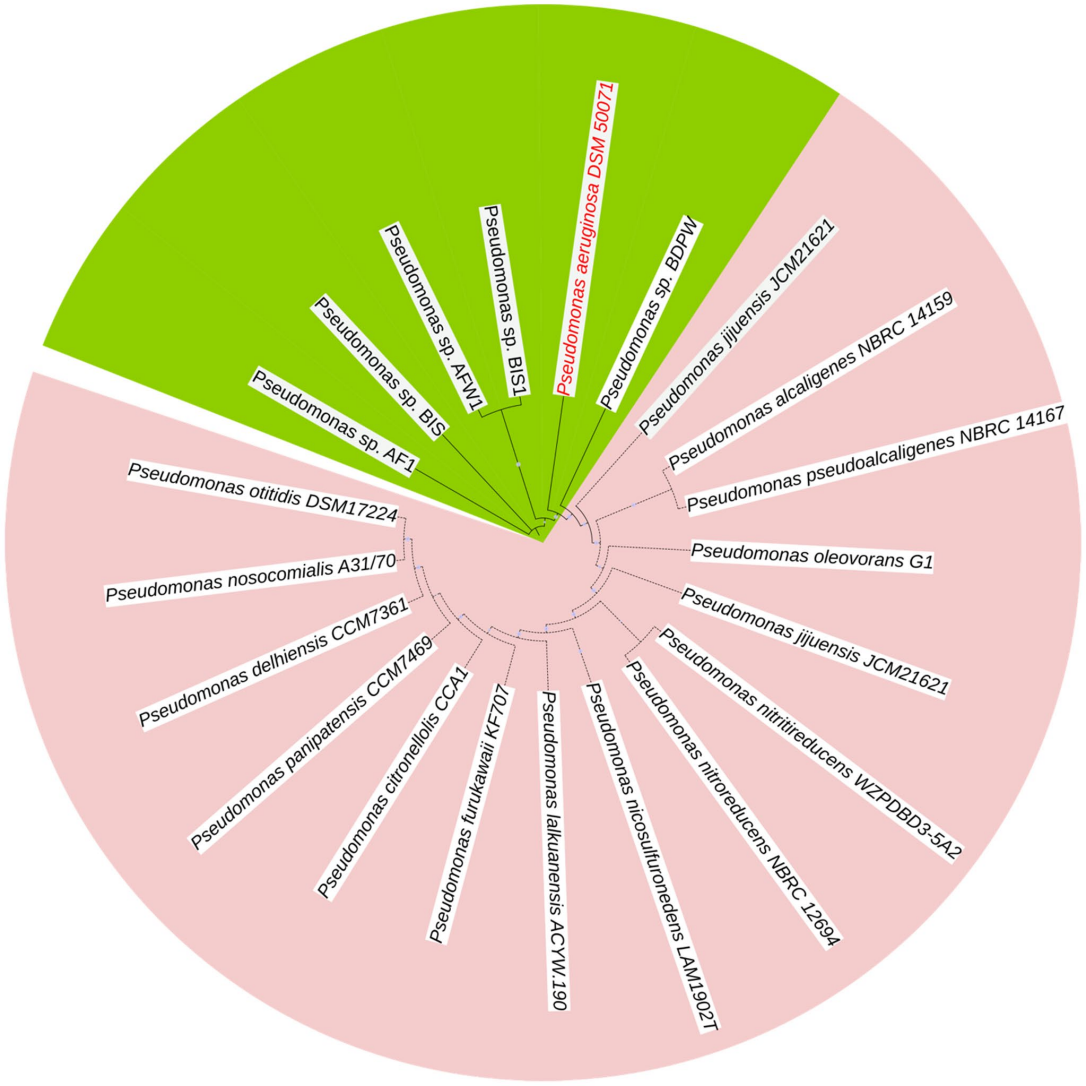
Dendrogram of *P. aeruginosa* isolates displaying comparisons of arrangements.

The dendrogram (Figure 1) displays the phylogenetic relationship between *Pseudomonas* isolates, highlighting the clustering and evolutionary relatedness among different strains. Genomic clustering revealed that all five isolates (*Pseudomonas* sp. AF1, *Pseudomonas* sp. BIS, *Pseudomonas* sp. AFW1, *Pseudomonas* sp. BIS1, and *Pseudomonas* sp. BDPW) formed a distinct clade closely aligned with *P. aeruginosa* DSM 50071, suggesting a shared lineage. The high bootstrap values (100%) at major nodes confirm the robustness and reliability of the clustering. The isolates clustered into a monophyletic group, indicating their close genetic relationship and potential classification within the *P. aeruginosa* species complex. Notably, *Pseudomonas* sp. AF1, AFW1, and BIS are positioned within the same sub-clade, indicating greater similarity among these strains compared to *Pseudomonas* sp. BWP and BDPW, which form a separate but closely related branch. The clustering of *Pseudomonas* sp. BIS1 near *Pseudomonas* sp. BIS further suggests a close genetic relationship, potentially indicating a common origin or similar ecological niche.

Outside this primary clade, other *Pseudomonas* species, such as *P. citronellolis* LMG 18378 and *P. alcaligenes* NBRC 14159, form distinct clades, confirming the genetic divergence between our isolates and these environmental *Pseudomonas* species. This reinforces that our isolates are genetically closer to *P. aeruginosa* DSM 50071 than to other *Pseudomonas* species.

The resulting tree revealed that the isolates clustered into two distinct sub-clusters. *Pseudomonas* sp. AFW1, *Pseudomonas* sp. BIS, and *Pseudomonas* sp. BIS1 formed a closely related clade, indicating high genetic similarity among these strains. The isolate from the swimming pool (*Pseudomonas* sp. BDPW) did not cluster with other isolates, suggesting it is genetically distinct from the other strains analyzed. This observation highlights the genetic diversity among the environmental *Pseudomonas* isolates.

### 3.2 Pre-processing, genome assembly, annotation, MLST and bioinformatics

Table 1 presents the general genome features of the five *P. aeruginosa* isolates. The coding sequences (CDS) vary significantly among the strains, with BIS1 showing the highest CDS count (6,638), while AF1 has the lowest (6,132). This variation may reflect differences in each strain’s functional capacities and environmental adaptability. The number of tRNA genes ranges from 42 in AF1 to 53 in BIS, suggesting potential differences in translation efficiency and stress adaptation mechanisms. Additionally, the presence of CRISPR repeats was noted in most isolates except for BDPW, which might indicate reduced immunity against horizontal gene transfer and increased susceptibility to bacteriophage infections. The variability in virulence factors, with BIS1 possessing the highest number (354) and BDPW the lowest (340), highlights the differing pathogenic potentials among the isolates. Antibiotic resistance genes were identified in all isolates, emphasizing their ability to survive in antimicrobial-rich environments (Table 1).

**Table 1:** Summary of genome features of 5 *P. aeruginosa* isolated sources in Sodwana Bay.

Genomic features	*Pseudomonas* sp. BDPW		*Pseudomonas* sp. BIS		*Pseudomonas* sp. BIS 1		*Pseudomonas* sp. AF1		*Pseudomonas* sp. AFW1	
	BV-BRC	GenBank/RefSeq	BV-BRC	GenBank/RefSeq	BV-BRC	GenBank/RefSeq	BV-BRC	GenBank/RefSeq	BV-BRC	GenBank/RefSeq
CDS	6,351	6,132	6,513	6,264	6,635	6,377	6,663	6,378	6,616	6,355
tRNA	51	50	53	52	51	50	44	42	46	45
Crispr_repeat	0	0	44	0	56	0	65	0	56	0
Crispr_spacer	0	0	42	0	54	0	62	0	54	0
Regulatory	8	8	8	8	8	8	8	8	8	8
Misc RNA	4	0	4	0	4	0	4	0	4	0
rRNA	3	3	3	3	3	3	3	3	3	3
Gene	0	6,095	0	6,215	0	6,313	0	6,324	0	6,305
Pseudogene	0	94	0	108	0	121	0	103	0	102
ncRNA	0	3	0	3	0	3	0	3	0	3
tmRNA	0	1	0	1	0	1	0	1	0	1

Specialty genes	Source	No. genes	Source	No. genes	Source	No. genes	Source	No. genes	Source	No. genes
Virulence factor	VFDB	248	VFDB	238	VFDB	256	VFDB	253	VFDB	247
Virulence factor	Victors	91	Victors	90	Victors	97	Victors	93	Victors	92
Virulence factor	PATRIC_VF	1	PATRIC_VF	1	PATRIC_VF	1	PATRIC_VF	2	PATRIC_VF	1
Transporter	TCDB	195	TCDB	188	TCDB	190	TCDB	193	TCDB	195
Drug target	Drug Bank	63	Drug Bank	64	Drug Bank	67	Drug Bank	66	Drug Bank	62
Drug target	TTD	9	TTD	11	TTD	11	TTD	9	TTD	9
Antibiotic resistance	CARD	53	CARD	53	CARD	54	CARD	55	CARD	54
Antibiotic resistance	NDARO	5	NDARO	5	NDARO	5	NDARO	6	NDARO	5
Antibiotic resistance	PATRIC	102	PATRIC	100	PATRIC	99	PATRIC	97	PATRIC	101

Table 2 summarizes the MLST profiles of the five *P. aeruginosa* isolates. Two major sequence types (ST244 and ST971) were identified. ST244, a globally recognized high-risk sequence type associated with multi-drug resistance, was detected in AF1 and BIS1. ST971, found in BDPW, is less frequently reported, indicating the possibility of a novel environmental sequence type with unique resistance mechanisms. The absence of the *aroE* gene in isolates AFW1 and BIS resulted in incomplete sequence typing, suggesting niche-specific adaptations or genetic drift in these isolates.

**Table 2:** MLST profiles of the five *Pseudomonas* sp. isolated from different sources.

Isolates	Organism	ST	Housekeeping genes						
*Pseudomonas* sp. AF1	*P. aeruginosa*	244	acsA(17)	aroE(5)	guaA(12)	mutL(3)	nuoD(14)	ppsA(4)	trpE(7)
*Pseudomonas* sp. AFW1	*P. aeruginosa*	–	acsA(17)	aroE(−)	guaA(12)	mutL(3)	nuoD(14)	ppsA(4)	trpE(7)
*Pseudomonas* sp. BDPW	*P. aeruginosa*	971	acsA(38)	aroE(8)	guaA(5)	mutL(67)	nuoD(2)	ppsA(40)	trpE(13)
*Pseudomonas* sp. BIS	*P. aeruginosa*	–	acsA(17)	aroE(−)	guaA(12)	mutL(3)	nuoD(14)	ppsA(4)	trpE(7)
*Pseudomonas* sp. BIS1	*P. aeruginosa*	244	acsA(17)	aroE(5)	guaA(12)	mutL(3)	nuoD(14)	ppsA(4)	trpE(7)

**Figure 2:**
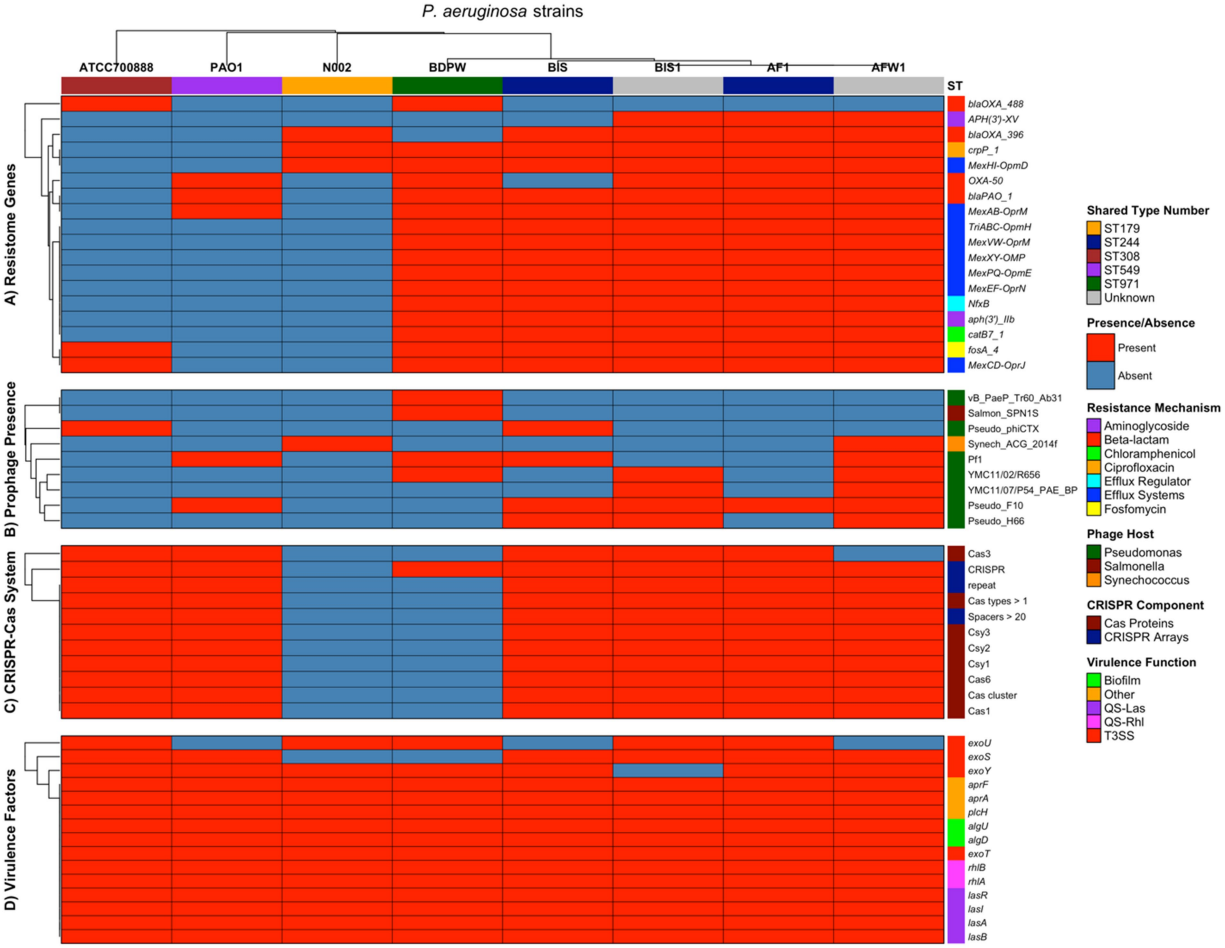
Multi-panel heatmap visualization of the comprehensive comparative analysis of four key genetic elements across our five *P. aeruginosa* strains (AF1, AFW1, BIS, BIS1, and BDPW) and three reference strains (PAO1, N002, and ATCC700888).

Although two isolates lacked ST values (unknown) due to missing allelic information, the results were visualized using heatmaps to provide a comparative overview of antimicrobial resistance patterns and clonal distribution. This approach allowed for the identification of distinct resistance profiles (resistome) and potential epidemiological links among the isolates. The multi-panel heatmap visualization (Figure 2) provides a comprehensive comparative analysis of four key genetic elements across our five *P. aeruginosa* strains (AF1, AFW1, BIS, BIS1, and BDPW) and three reference strains (PAO1, N002, and ATCC700888). Panel A of the heatmap showing the presence or absence of resistome in the 8 strains revealed a striking dichotomy between our five strains and the other strains used as control for comparison. Our five strains exhibit an extensive antimicrobial resistance arsenal, possessing 83.3–94.4% of the evaluated resistance genes. In contrast, the reference strains (PAO1, N002, and ATCC700888) contain merely 16.7% of these determinants each. Of particular significance, all five strains harbour a comprehensive suite of efflux pump systems (*MexAB-OprM, MexCD-OprJ, MexEF-OprN, MexPQ-OpmE, MexXY-OMP, MexVW-OprM,* and *TriABC-OpmH*), which confer resistance to multiple antibiotic classes. The aminoglycoside resistance gene *aph(3′)-IIb* was universally present in the 5 strains but absent in the selected control strains. Notably, ST244 isolates (AF1 and BIS) show a distinctive resistance profile, with BIS lacking *aph(3′)-XV* while maintaining *blaPAO_1*, a pattern differentiating it from other strain in our study.

The beta-lactamase distribution showed interesting patterns, with *blaOXA_488* uniquely present in BDPW (ST971) and ATCC700888, while *blaOXA_396* was found in all environmental/clinical strains. This suggests evolutionary divergence or differential selective pressures acting on beta-lactamase acquisition. This clustering visualization underscores the variability in antibiotic resistance profiles, suggesting possible differences in the mechanisms of resistance among the strains. These findings offer valuable insights into the resistome diversity within *Pseudomonas* species, potentially guiding antibiotic stewardship and therapeutic decision-making.

Panel B of the heatmap showing prophage presence revealed a remarkable variation of prophage presence across the strains, with environmental isolates harboring significantly more prophages than reference strains. AFW1 displays the most extensive prophage repertoire (6 prophages, 66.7%), followed by BIS and BIS1 (4 prophages each, 44.4%). The reference strains contain only 1–2 prophages each. The Pseudo_F10 prophage appears to be the most predominant in the environmental/clinical isolates (present in AF1, AFW1, BIS, BIS1) and PAO1, but absent in BDPW and the other two control strains. Conversely, BDPW exhibits a unique prophage profile, harboring Salmon_SPN1S (a Salmonella phage) and vB_PaeP_Tr60_Ab31, which are absent in all other isolates. The Synechococcus phage (Synech_ACG_2014f) is uniquely present in AFW1 and N002, suggesting potential horizontal gene transfer from cyanobacteria. The prophage distribution patterns suggest distinct evolutionary trajectories or environmental exposures among the isolates, with AFW1 potentially serving as a significant reservoir for phage-mediated horizontal gene transfer. Most strains contained PHAGE_Pseudo_F10, while PHAGE_Pseudo_YMC11/07/P54_PAE_BP was the least common. These prophage elements encode various structural and functional proteins, such as integrase, terminase, and transposase, which may contribute to the isolates’ adaptability and virulence.

Panel C of the heatmap showing CRISPR-Cas System revealed a nearly binary pattern: almost complete presence (90.9–100%) in all isolates except BDPW (9.1%) and N002 (0%). This suggests that BDPW has a highly reduced CRISPR-Cas system, maintaining only the CRISPR array itself without the associated Cas proteins, while N002 completely lacks this defense system. The complete absence of CRISPR-Cas in N002 may explain its susceptibility to acquiring the unique Synechococcus phage observed in the prophage panel. Conversely, the well-developed CRISPR-Cas systems in the other isolates likely provide robust defense against foreign genetic elements, potentially limiting prophage acquisition despite environmental exposure. Interestingly, the ST244 isolates (AF1 and BIS) and reference strains PAO1 and ATCC700888 possess identical CRISPR-Cas profiles despite their otherwise divergent genetic backgrounds, suggesting conservation of this defense system across distantly related lineages. AF1 had the most complex CRISPR-Cas system with six distinct loci and several Cas proteins, suggesting a high level of defense against horizontal gene transfer. In contrast, BDPW showed only one CRISPR locus with no associated Cas genes, possibly explaining its unique resistance traits compared to the other isolates.

Panel D of the heatmap showing the virulence factors (genes) revealed a very high conservation for all the analyzed genetic elements evaluated (93.3–100% presence), indicating their fundamental importance to *P. aeruginosa* pathogenicity regardless of isolation source or ST values. Notably, the distribution of Type III Secretion System (*T3SS*) effectors follows previously described mutually exclusive patterns: *exoU* and *exoS* generally do not co-occur. AF1, BIS1, BDPW, and ATCC700888 harbour *exoU*, associated with heightened virulence and acute cytotoxicity, while BIS, AFW1, and PAO1 contained *exoS*, linked to invasiveness and chronic infections. This pattern suggests potential adaptation to different infection strategies in *P. aeruginosa* strains. The quorum sensing systems (*QS-Las* and *QS-Rhl*) are universally conserved across all strains, emphasizing their fundamental role in *P. aeruginosa* pathobiology and coordinate regulation of virulence. Interestingly, we analyzed virulence genes associated with key pathogenic mechanisms, including alginate biosynthesis and regulation, rhamnolipid biosynthesis, iron uptake, quorum sensing, protease, and toxin production, using the Virulence Factor Database (VFDB). These virulence factors are particularly relevant to infections of the urinary tract and lungs. We also included the virulence genes of the PAO1 strain as a reference, incorporating the *exoU* gene of the ATCC700888 strain as a comparison point. We examined genes coding for type 3 secretion system effectors, including *exoS*, *exoT*, *exoU*, and *exoY*. The clustering algorithm enabled visualization of patterns across the genomes studied, highlighting that *exoS* was commonly found across most genomes, excluding *P. aeruginosa* BDPW. Meanwhile, *exoU* appeared solely in the genomes of *P. aeruginosa* AF1 and BDPW, and *exoY* and *exoT* were universally present in all genomes analyzed. Remarkably, the *exoS* and *exoU* genes, typically mutually exclusive, co-occurred in *P. aeruginosa* AF1, marking a unique observation in our dataset. More so, protease genes such as *lasA* (elastase A) and *lasB* (elastase B) were consistently present across all strains. The *aprA* gene, encoding alkaline protease, and *plcH*, a phospholipase gene, were also ubiquitously found, underscoring their widespread role in *Pseudomonas* pathogenicity. This clustering analysis visually underscores the differential presence of virulence determinants, offering insights into potential pathogenic strategies across *Pseudomonas* species and informing targeted therapeutic approaches.

Statistical analysis revealed distinct patterns of genomic diversity among the eight *P. aeruginosa* isolates. Jaccard similarity indices demonstrated high genomic relatedness among our strains (similarity indices 0.83–1.00 for resistome genes), particularly between isolates with the same shared type number (ST244: AF1 and BIS), while the strains used as control showed substantial divergence (similarity indices 0.00–0.19).

The five strains possessed substantially more resistance determinants compared to the control strains. The Jaccard similarity indices between these groups range from 0.11–0.20, indicating minimal resistome overlap. Thus, supporting evidence to suggest acquisition of multiple resistance determinants (likely through horizontal gene transfer or selective pressure) in their respective environments from whence they were isolated (ocean bottom and swimming pool). Similarly, the five strains harbour more diverse prophage repertoires (1–6 prophages) compared to control strains. The prophage profiles show minimal overlap between groups (Jaccard indices 0.00–0.50), indicating distinct exposure histories or lysogeny events. Except for strain N002, the CRISPR-Cas systems are highly conserved between the environmental and the control strains PAO1 and ATCC700888 (Jaccard indices 0.91–1.00) which are highly pathogenic. This suggests strong selective pressure to maintain this defense system across diverse *P. aeruginosa* lineages.

The virulence determinants show remarkable conservation between our strains and the controls (Jaccard indices 0.87–1.00), underscoring their essential role in *P. aeruginosa* fitness regardless of isolation source.

The overall genomic profiles indicate that while the strains obtained from swimming pool, and ocean bottom maintain the core virulence arsenal of the control strains, they have acquired additional genetic elements, particularly resistance determinants and prophages, that may confer adaptations to specific environmental niches or clinical settings. The ST244 isolates (AF1 and BIS) display particularly high genetic similarity despite potentially different isolation sources (with AFI from ocean bottom and BIS from otitis externa of infected scuba diver), suggesting clonal dissemination of this successful lineage.

Hierarchical clustering based on all genetic features identified three major clusters (Supplementary Figure S3): our *Pseudomonas* strains (AF1, AFW1, BIS, BIS1), the ST971 isolate (BDPW) in an intermediate position, and reference strains (PAO1, N002, ATCC700888) forming a separate cluster. Correlation analysis between genetic categories revealed a positive correlation between resistome and prophage content (r = 0.67), suggesting potential co-acquisition of these elements. At the same time, virulence factors showed a negative correlation with prophage presence (r = −0.61), indicating possible antagonistic relationships between these genomic features. The CRISPR-Cas systems showed a moderate positive correlation with virulence factors (r = 0.35). Feature richness analysis demonstrated that while virulence factors were largely conserved across all isolates (mean = 14.25 ± 0.46), other genetic elements displayed substantial variation, particularly prophage content (mean = 2.88 ± 1.89) and resistome genes (mean = 11.38 ± 6.97) (Supplementary material).

## 4 Discussion

This study provides comprehensive genomic insights into five *P. aeruginosa* isolates from Sodwana Bay, South Africa, revealing their close alignment with strain DSM 50071 and confirming their classification within this species complex. The high bootstrap values (100%) at major nodes demonstrated the robustness of this clustering, with AF1, AFW1, and BIS showing greater genetic similarity compared to BIS1 and BDPW thus highlighting the genetic diversity within environmental *P. aeruginosa* populations. Our molecular identification confirmed all isolates as *P. aeruginosa* through PCR amplification of species-specific 16S rRNA gene regions, providing a reliable foundation for subsequent genomic analyses (Madaha et al., 2020). The clustering of these isolates into two distinct subgroups, with AF1, AFW1, and BIS forming a closely related clade and BDPW standing apart, underscores the genetic variability present even within a single environmental niche. This observation aligns with previous findings that environmental *P. aeruginosa* populations can be highly diverse and may serve as reservoirs for clinically relevant lineages (Khan et al., 2008; Pirnay et al., 2009).

### 4.1 Sequence types and genetic diversity

The MLST analysis identified two major sequence types (ST244 and ST971) among our five isolates. ST244, a globally recognized high-risk clone, is frequently associated with multidrug resistance and has been implicated in clinical outbreaks worldwide including hospital-acquired pneumonia, cystic fibrosis, urinary tract infections, and bloodstream infections (Maatallah et al., 2011; Chen et al., 2014; Slekovec et al., 2019; Salem et al., 2024; de Sousa et al., 2025). The detection of ST244 in marine isolates suggests that environmental reservoirs may play a crucial role in the persistence and dissemination of high-risk clones, echoing recent reports of environmental sources contributing to the spread of antimicrobial resistance (Salem et al., 2024). The presence of identical ST244 isolates from ocean sediment (AF1) and human infection (BIS from otitis externa) strongly indicates potential transmission between environmental and clinical settings, highlighting the importance of ecological surveillance for clinically relevant strains. In contrast, ST971 (found in BDPW) has been less frequently reported, with limited data on its resistance patterns and virulence potential. Its unique positioning in hierarchical clustering between our leading isolate group and the reference strains suggests it may represent an evolutionary intermediate or a strain adapting to specific environmental niches. The identification of ST971 in this study may indicate the presence of novel environmental STs harboring previously uncharacterized resistance mechanisms.

The absence of the *aroE* gene in two isolates (AFW1 and BIS) resulted in incomplete sequence typing, suggesting potential niche-specific adaptations or genetic drift. This gene loss phenomenon aligns with previous observations of auxotrophy in clinical isolates, where strains in nutrient-dense environments may depend on ambient absorption rather than endogenous production (Silva-Pereira et al., 2024). Such genomic adaptations highlight the dynamic nature of *P. aeruginosa* evolution in response to environmental pressures.

### 4.2 Genomic features and resistome (antibiotic resistance) analysis

The genome assembly sizes ranged from 6.3–6.8 Mbp with considerable variation in coding sequences (6,132–6,638) and tRNA genes (42–53) among isolates, reflecting functional capacities and environmental adaptability differences. The correlation between higher CDS counts in isolates like BIS1 and increased virulence factor presence (354 factors) suggests enhanced genetic potential for adaptation and pathogenicity. Our resistome analysis demonstrated that the five environmental isolates possess an extensive array of antimicrobial resistance genes, including those encoding for efflux pumps (*MexAB-OprM, MexCD-OprJ, MexEF-OprN, MexPQ-OpmE, MexXY-OMP, MexVW-OprM, and TriABC-OpmH*) and aminoglycoside resistance genes such as *aph(3′)-IIb* conferring resistance to various antibiotic classes. The aminoglycoside resistance gene *aph(3′)-IIb* was universally present in our isolates but absent in control strains, suggesting possible environmental selection for this resistance determinant. The *β*-lactamase distribution showed interesting patterns, with *blaOXA_488* uniquely present in BDPW (ST971) and *blaOXA_396* found in all other environmental isolates. Both are variants of oxacillinase *blaOXA-50*, capable of hydrolyzing carbapenems in addition to other β-lactams (Castanheira et al., 2021). These carbapenemase-producing isolates emphasize the role of environmental reservoirs in the potential dissemination of resistance genes. Previous studies have reported outbreaks of *OXA-488-*producing organisms worldwide, particularly in the Middle East and Northern Africa (Dautzenberg et al., 2014; Fernandez et al., 2015; Manenzhe et al., 2015; Guo et al., 2016; Al-Orphaly et al., 2021). This broad resistome profile mirrors that of clinical multidrug-resistant (MDR) and extensively drug-resistant (XDR) strains, as previously reported in global surveillance studies (Kos et al., 2015; Salem et al., 2024). Notably, the presence of carbapenemase genes such as *blaOXA-488* in BDPW and *blaOXA-396* in other isolates indicates ongoing evolution and diversification of beta-lactamase determinants, which are critical for carbapenem resistance as mutations in porin genes, particularly *oprD*, have been shown to contribute significantly to carbapenem and colistin resistance in *P. aeruginosa* (Kos et al., 2015; Tran et al., 2024). The high prevalence of these resistance determinants in environmental isolates underscores the risk of resistance gene dissemination from non-clinical settings.

### 4.3 Prophages and CRISPR-Cas systems

The prophage profiling demonstrated a higher diversity and abundance of prophage elements our five environmental isolates compared to control strains. The presence of diverse prophages, including Pseudo_F10 and Salmon_SPN1S, suggests that horizontal gene transfer via phage-mediated mechanisms may significantly shape the genomic landscape and adaptability of environmental *P. aeruginosa* (Salem et al., 2024). This is further supported by the observed positive correlation between resistome and prophage content, indicating potential co-acquisition of these elements. AFW1 displayed the most extensive prophage repertoire (6 prophages, 66.7%), suggesting it may serve as a significant reservoir for phage-mediated horizontal gene transfer. The Pseudo_F10 prophage was predominant in most isolates (AF1, AFW1, BIS, BIS1) but absent in BDPW, which exhibited a unique prophage profile including Salmon_SPN1S. These distinct prophage patterns suggest different evolutionary trajectories or environmental exposures among the isolates. The positive correlation between resistome and prophage content (r = 0.67) suggests potential co-acquisition of these elements, possibly through horizontal gene transfer events that simultaneously introduce both resistance genes and prophages. This co-acquisition phenomenon may contribute to the rapid adaptation of *P. aeruginosa* to new environment and antimicrobial challenges. The CRISPR-Cas system analysis revealed near-complete presence in all isolates except BDPW (9.1%) and reference strain N002 (0%). This suggests BDPW has a highly reduced CRISPR-Cas system, maintaining only the CRISPR array without associated *Cas* proteins. The absence or reduction of CRISPR-Cas systems in specific isolates may explain their unique genetic compositions, as these strains would be more susceptible to acquiring foreign genetic elements (possibly through horizontal gene transfer) such as prophages and resistance genes as observed in the report by Salem et al. (2024). This alignment with previous research suggests that CRISPR-Cas systems serve as an adaptive immune response to mobile genetic elements, particularly bacteriophages (Barrangou et al., 2007; Horvath and Barrangou, 2010; Barrangou and Marraffini, 2014) and resistome.

### 4.4 Virulence factors and pathogenicity

Virulence gene analysis showed remarkable conservation across all isolates, with 93.3–100% of evaluated virulence factors present in all the evaluated strains in the study indicating their fundamental importance to *P. aeruginosa* pathogenicity regardless of isolation source. The distribution of Type III Secretion System (T3SS) effectors followed established mutually exclusive patterns, with *exoU* and *exoS* rarely co-occurring (Chowdhury et al., 2023). The presence of both acute (*exoU*) and invasive (*exoS*) virulence determinants highlights the pathogenic potential of these environmental isolates and their capacity to cause a range of infections, consistent with findings from both clinical and environmental studies (Chowdhury et al., 2023; Salem et al., 2024). The universal presence of quorum sensing and biofilm-associated genes further emphasizes the adaptability and persistence of *P. aeruginosa* in various ecological niches. AF1, BIS1, BDPW, and reference strain ATCC700888 harboured *exoU*, associated with heightened virulence and cytotoxicity, while BIS, AFW1, and PAO1 contained *exoS*, linked to invasiveness and chronic infections. Remarkably, *P. aeruginosa* AF1 uniquely possessed both *exoU* and *exoS* genes, which are typically mutually exclusive, representing a potentially rare genotype with expanded virulence capabilities. All isolates possessed genes for proteases (*lasA, lasB*), alkaline protease (*aprA*), and phospholipase (*plcH*), underscoring their widespread role in *Pseudomonas* pathogenicity. The quorum sensing systems (*QS-Las* and *QS-Rhl*) were universally conserved across all strains, emphasizing their fundamental role in coordinating virulence expression. The moderate positive correlation between CRISPR-Cas systems and virulence factors (r = 0.35) suggests potential regulatory interactions between these genomic features, aligning with previous findings that CRISPR-Cas systems can influence biofilm formation and virulence expression in *Pseudomonas* (Zegans et al., 2009; Cady and O'Toole, 2011). Our study demonstrated that environmental *P. aeruginosa* isolates can harbour resistance and virulence profiles comparable to those of clinical strains, thus, highlighting the potential for environmental sources to contribute to the emergence and spread of high-risk clones. The convergence of high-risk sequence types, multidrug resistance, and conserved virulence factors in environmental isolates underscores the need for integrated surveillance and control strategies that encompass both clinical and environmental contexts (Kos et al., 2015; Chowdhury et al., 2023; Salem et al., 2024).

### 4.5 Ecological and public health implications

Identifying clinically significant sequence types (particularly ST244) in environmental samples highlights the interconnectedness of environmental and clinical reservoirs of *P. aeruginosa*. The high Jaccard similarity indices (0.83–1.00) for resistome genes among our environmental isolates, compared to substantial divergence from control strains (0.00–0.19), suggests the recent acquisition of multiple resistance determinants, likely through horizontal gene transfer or selective pressure in their respective environments. These findings contribute to Sustainable Development Goal 3 by underscoring the urgent need for innovative antimicrobial strategies to manage multidrug-resistant pathogens. The prevalence of carbapenemase-producing strains within environmental isolates highlights the potential for environmental reservoirs to serve as sources of resistance genes, which could eventually impact public health. Identifying similar genetic profiles between environmental and clinical isolates reinforces the One Health concept, emphasizing that human, animal, and environmental health are interconnected, and addressing antimicrobial resistance requires integrated surveillance across these domains.

### 4.6 Limitations of the study

The small number of isolates analyzed, which may not fully represent the genetic diversity of *P. aeruginosa* in marine settings is a limitation of our study. Although genomic predictions of resistance, virulence, and mobile elements were conducted, phenotypic validation was not performed. In addition, while insertion sequences and genomic islands were identified bioinformatically, experimental confirmation of horizontal gene transfer was not undertaken. Future studies involving larger sample sizes and functional assays are needed to strengthen these findings.

## 5 Conclusion

Our study offers valuable genomic insights into five multidrug-resistant *P. aeruginosa* isolates obtained from Sodwana Bay, South Africa. The isolates exhibited notable genetic diversity, clustering closely with the high-risk ST244 genotype and presenting a wide range of resistance and virulence factors. Significant findings include the identification of carbapenem-resistant genes (such as the *blaOXA-50* and *blaOXA-488*) variation, virulence gene distributions (such as *exoS*, and *exoU*), and distinct CRISPR-Cas profiles across the isolates. The presence of horizontally acquired resistance determinants and the clustering of isolates with clinical strains underscores the role of environmental reservoirs in the potential dissemination of antimicrobial resistance. These findings highlight the critical need for robust monitoring strategies to mitigate the risk of resistance transfer and the emergence of high-risk clones. This research contributes to Sustainable Development Goal 3 and One Health by advancing our understanding of antimicrobial resistance in environmental settings and guiding the development of targeted therapeutic and containment strategies. Further studies are warranted to explore the clinical implications of these findings and their impact on public health.

## Data Availability

The datasets presented in this study can be found in online repositories. The names of the repository/repositories and accession number(s) can be found in the article/Supplementary material.

## Footnotes

^1^ https://usegalaxy.org

^2^ https://pubmlst.org/paeruginosa/

^3^ https://www.bv-brc.org/view/Taxonomy/2806610#view_tab=specialtyGenes
