# Supplementary material for: Genomic insights into five selected multidrug-resistant Pseudomonas aeruginosa isolated from Sodwana Bay, South Africa
Source: Front Microbiol. 2025 Jul 2;16:1578578. doi: 10.3389/fmicb.2025.1578578 (PMC12263573; doi:10.3389/fmicb.2025.1578578)
Supplement: Supplementary file 1 [file Data_Sheet_1.docx]

Supplementary Materials


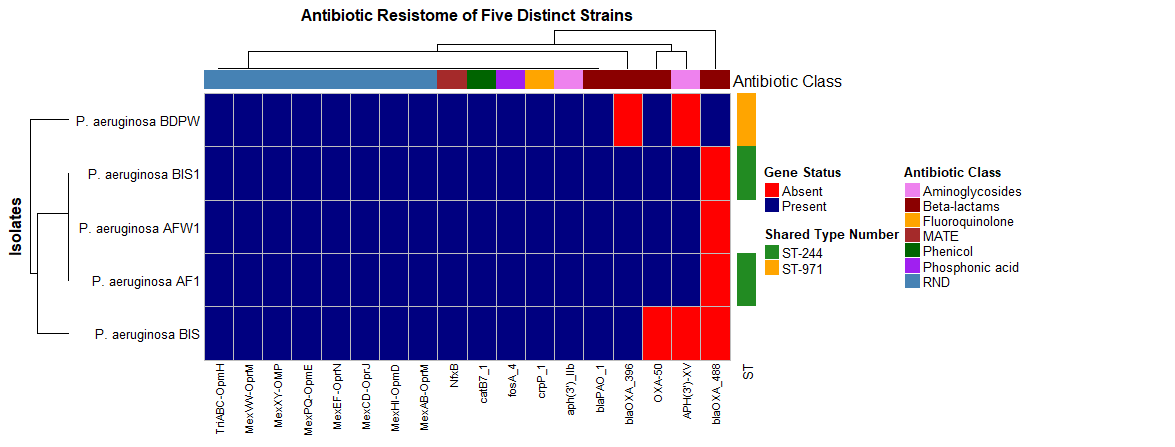


Figure S1. Heatmap of Resistome Profiles in *Pseudomonas aeruginosa* strains isolated from different sources

RND (Resistance-Nodulation-Division), MATE (Multidrug And Toxic compound Extrusion).

Table 3: Prophage-associated sequences present in the genomes of the 5 identified *Pseudomonas aeruginosa* strains

| Isolate | ST | Region | Completeness (Score) | The first and most common phage |
| --- | --- | --- | --- | --- |
| ***Pseudomonas aeruginosa* AF1** | 244 | 1 | Intact (107) | PHAGE_Pseudo_F10 |
| ***Pseudomonas aeruginosa* BDPW** | 971 | 1 | Incomplete (60) | PHAGE_Pseudo_YMC11/02/R656 |
|  |  | 2 | Intact (96) | PHAGE_Pseudo_Pf1 |
|  |  | 3 | Intact (150) | PHAGE_Pseudo_vB_PaeP_Tr60_Ab31 |
|  |  | 4 | Incomplete (20) | PHAGE_Salmon_SPN1S |
| ***Pseudomonas aeruginosa* BIS1** | 244 | 1 | Intact (150) | PHAGE_Pseudo_YMC11/02/R656 |
|  |  | 2 | Intact (102) | PHAGE_Pseudo_F10_ |
|  |  | 3 | Intact (120) | PHAGE_Pseudo_H66 |
|  |  | 4 | Questionable (70) | PHAGE_Pseudo_YMC11/07/P54_PAE_BP |
| ***Pseudomonas aeruginosa* AFW1** | - | 1 | Intact (107) | PHAGE_Pseudo_Pf1_NC_ |
|  |  | 2 | Intact (97) | PHAGE_Pseudo_F10 |
|  |  | 3 | Intact (150) | PHAGE_Pseudo_YMC11/02/R656 |
|  |  | 4 | Intact (100) | PHAGE_Pseudo_H66 |
|  |  | 5 | Incomplete (40) | PHAGE_Synech_ACG_2014f |
|  |  | 6 | Incomplete (30) | PHAGE_Pseudo_YMC11/07/P54_PAE_BP |
| ***Pseudomonas aeruginosa* BIS** | - | 1 | Intact (100) | PHAGE_Pseudo_F10 |
|  |  | 2 | Intact (100) | PHAGE_Pseudo_phiCTX |
|  |  | 3 | Questionable (80) | PHAGE_Pseudo_H66 |
|  |  | 4 | Questionable (75) | PHAGE_Pseudo_Pf1 |

**Key: Completeness (Score); Intact (score >90), Questionable (Score 70-90), Incomplete (Score <70)**

Table 4. CRISPR and CRISPR-associated protein (CRISPR-cas) system in the isolated *P. aeruginosa.*

| Isolate code | Shared Type | Region | Element | Number of spacers | Number of cas Genes (cas type) | Direct Repeat (DR) consensus/cas Gene |
| --- | --- | --- | --- | --- | --- | --- |
| AFW1 | - | 1 | CRISPR | 32 |  | TTTCTTAGCTGCCTACACGGCAGTGAAC |
|  |  |  | Cas cluster |  | 4(IF) | *Cas*6, *Csy*1, *Csy*2, *Csy*3 |
|  |  | 2 | CRISPR |  |  | GGCCAAAGAACTGGAGGGCAAGGTATGA |
|  |  |  | Cas cluster |  | 1(IF) | *Cas*1 |
| AF1 | 244 | 1 | CRISPR | 20 |  | GTTCACTGCCGTGTAGGCAGCTAAGAAA |
|  |  |  | Cas cluster |  | 6(IF) | *Cas*1, *Cas*3-*Cas*2, *Cas*6, *Csy*1, *Csy*2, *Csy*3 |
|  |  | 2 | CRISPR | 21 |  | TTTCTTAGCTGCCTATACGGCAGTGAAC |
|  |  | 3 | CRISPR | 2 |  | CTCATCCGCGACATCGGCGGATAACGCCCATGGCGTTATTCGCCCT |
|  |  |  | Cas cluster |  | 1(IF) | *Cas*3 |
|  |  | 4 | CRISPR | 32 |  | GTTCACTGCCGTGTAGGCAGCTAAGAAA |
|  |  |  | Cas cluster |  | 2(IF) | *Cas*1, *Cas*3-*Cas*2 |
|  |  | 5 | CRISPR | 21 |  | GTTCACTGCCGTATAGGCAGCTAAGAAA |
|  |  |  | Cas cluster |  | 3(IF) | *Cas*6, *Csy*2, *Csy*3 |
|  |  | 6 | CRISPR | 8 |  | GTTCACTGCCGTGTAGGCAGCTAAGAAA |
| BDPW | 971 | 1 | CRISPR | 1 | 0(IF) | GGCCAAAGAACTGGAGGGCAAGGTATGA |
| BIS | - | 1 | CRISPR | 20 |  | GTTCACTGCCGTGTAGGCAGCTAAGAAA |
|  |  |  | Cas cluster |  | 6(IF) | *Cas*1, *Cas*3-*Cas*2, *Cas*6, *Csy*1, *Csy*2, *Csy*3 |
|  |  | 2 | CRISPR | 21 |  | TTTCTTAGCTGCCTATACGGCAGTGAAC |
|  |  | 3 | CRISPR | 1 |  | GGCCAAAGAACTGGAGGGCAAGGTATGA |
| BIS 1 | 244 | 1 | CRISPR | 32 |  | GTTCACTGCCGTGTAGGCAGCTAAGAAA |
|  |  |  | Cas cluster |  | 6(IF) | *Cas1*, *Cas*3-*Cas*2, *Cas*6, *Csy*1, *Csy*2, *Csy*3. |
|  |  | 2 | CRISPR | 21 |  | TTTCTTAGCTGCCTATACGGCAGTGAAC |

Statistical Analysis

Figure S2-S9. Statistical Output of 5 *P. aeruginosa* strains and the controls used in the study.


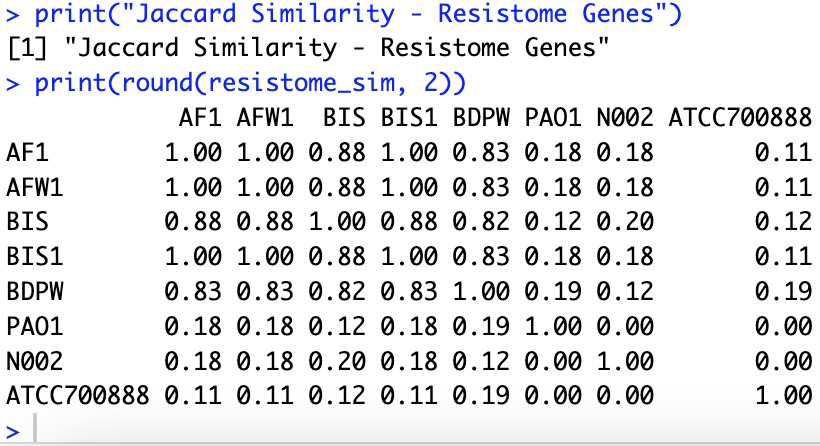


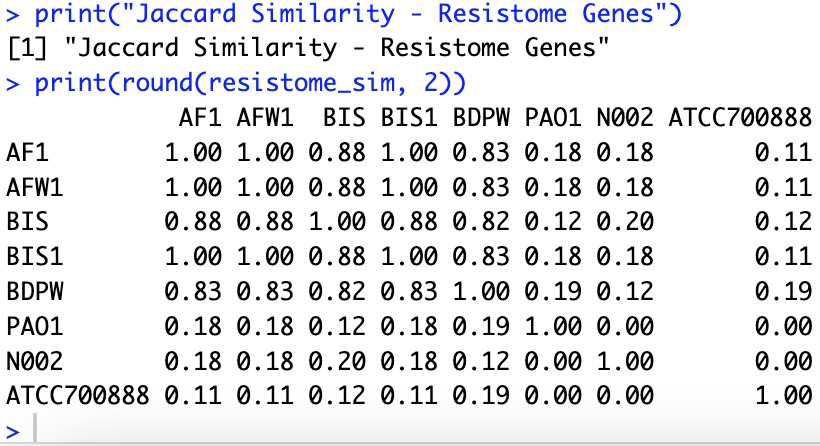


Figure S2 & S3. Jacard Similarity output for Resistome analysis.


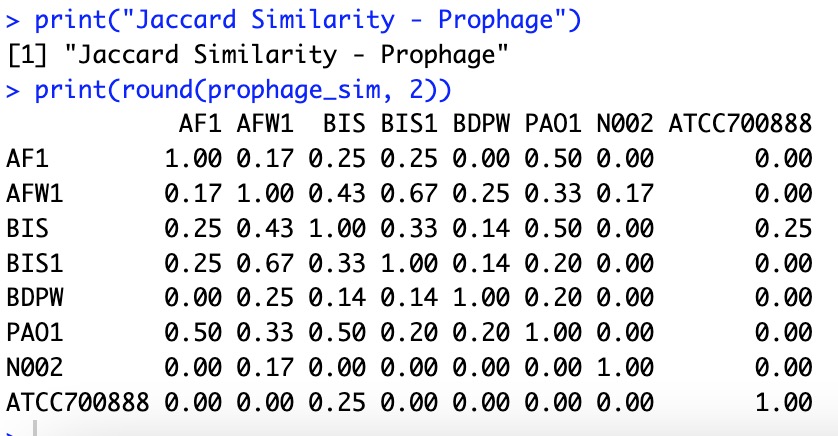


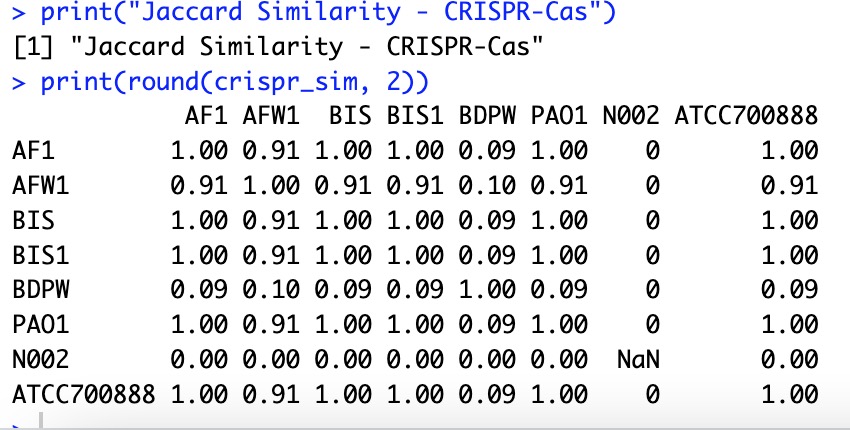


Figure S4 & S5. Jacard Similarity output for Prophage and CRISPAR analysis.


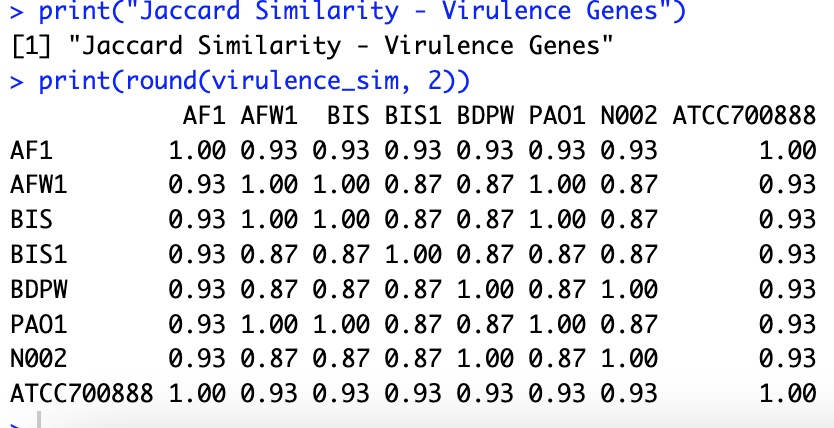


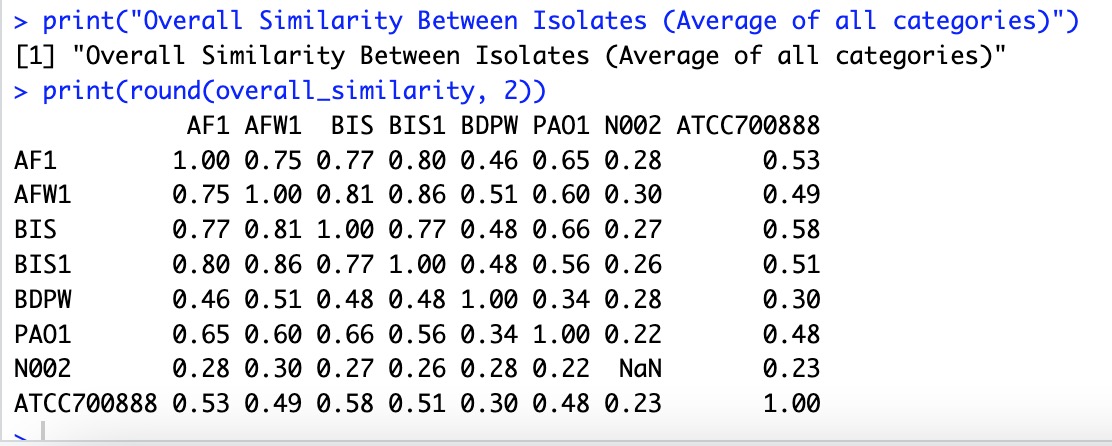


Figure S6 & S7. Jacard Similarity output for Virulence gene analysis and Overall similarity between isolates


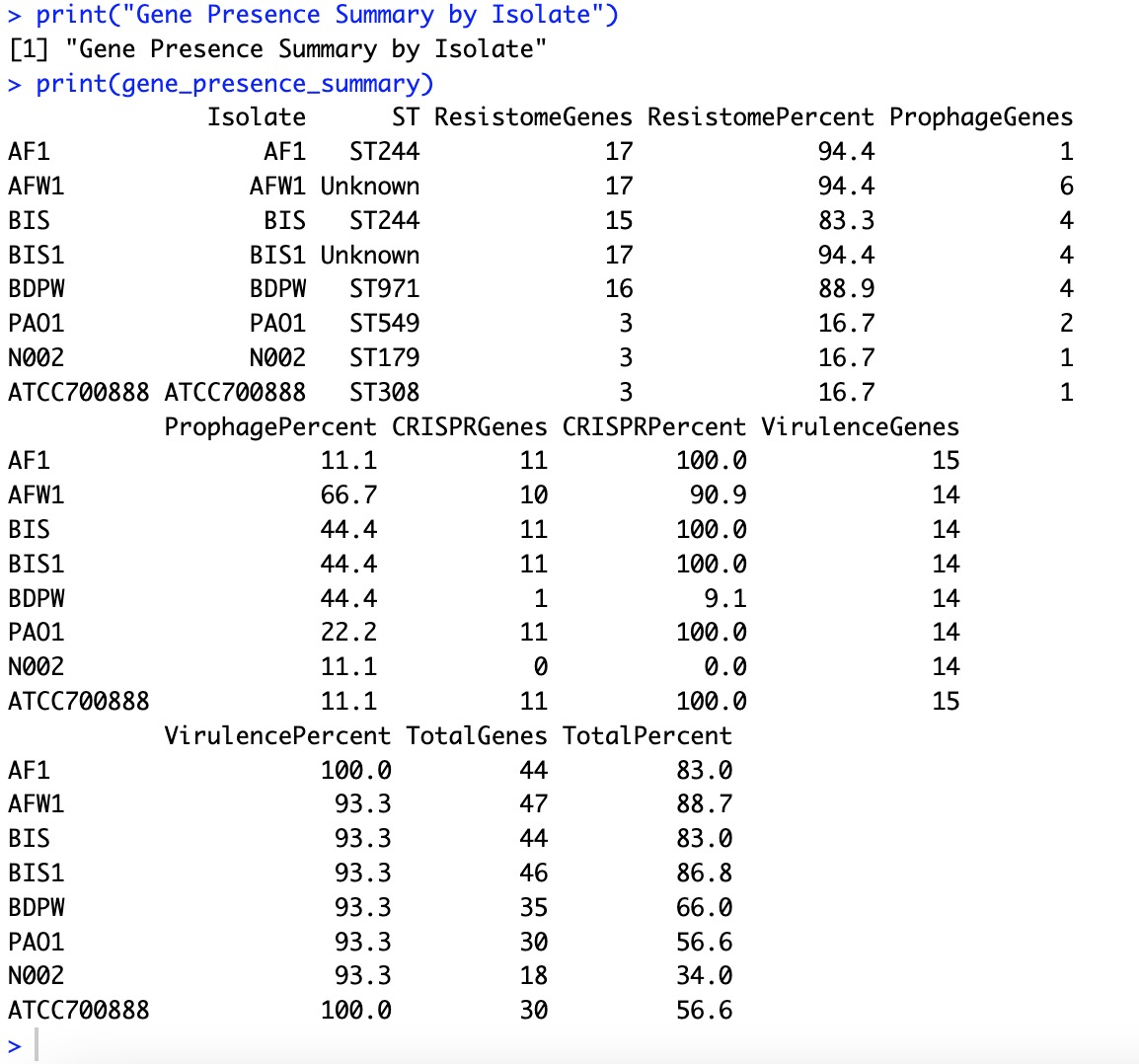


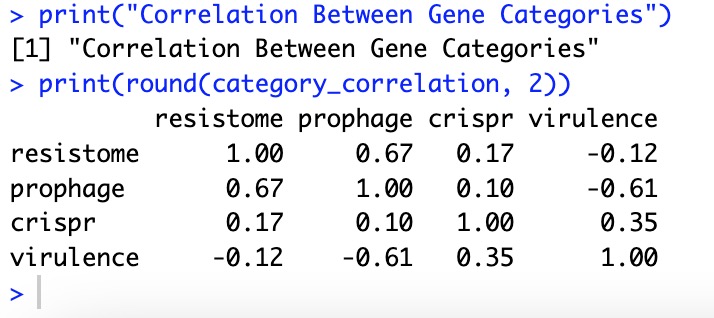


Figure S7 & S8. Summary of gene presence and correlation between isolates


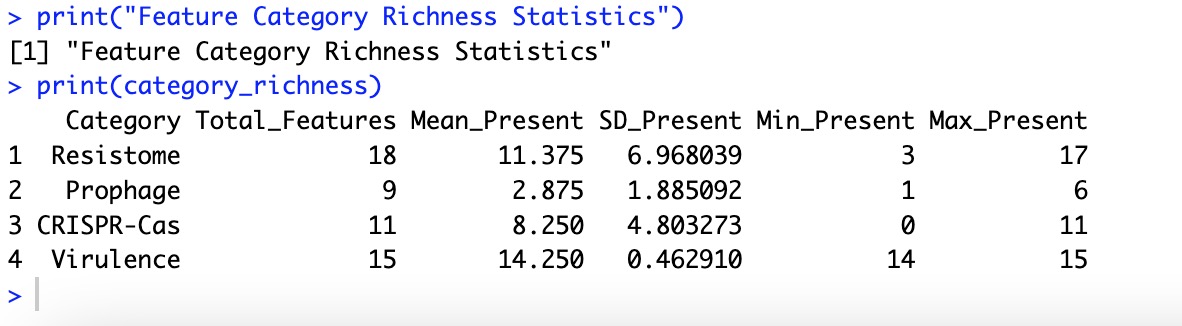


Figure S9. Richness statistics for all factors evaluated.


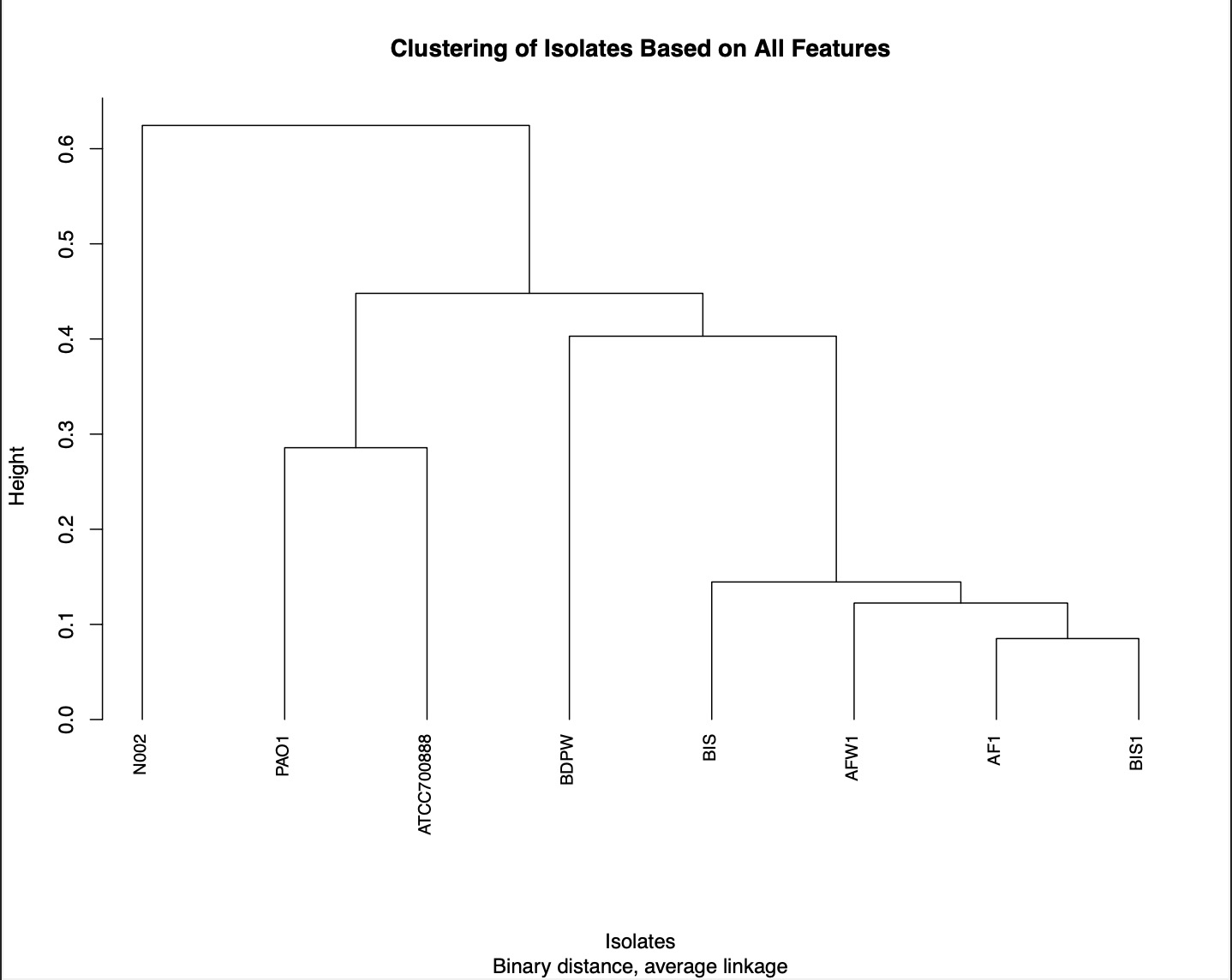


Figure S10. Clustering of all Isolates in the Study
